# Mouse genomic screen reveals novel host regulator of metastasis

**DOI:** 10.1186/s13059-017-1170-x

**Published:** 2017-02-16

**Authors:** Toni Celià-Terrassa, Yibin Kang

**Affiliations:** Department of Molecular Biology, Princeton University, Princeton, NJ 08544 USA

## Abstract

Tumor cells have to overcome challenges in the host tissue microenvironment to metastasize successfully to distant organs. In a recent *Nature* study, a genome-wide functional screen demonstrated that deficiency of the sphingosine-1-phoshate (S1P) transporter gene *Spns2* in endothelium increased immune-mediated cell killing by T cells and natural killer (NK) cells, thereby suppressing metastatic colonization.

Metastasis is a highly inefficient process—less than 0.02% of disseminated tumor cells (DTCs) are believed to be capable of seeding secondary tumors. Much of the high rate of attrition of DTCs occurs during the colonization step, where the arriving DTCs face a challenging microenvironment that is often distinctively different from that of the primary tumor [1]. Application of transcriptomic profiling and genome-wide functional screening strategies based on RNA interference, CRISPR-Cas9 genome editing, or transposon mutagenesis technologies have led to the discovery of various tumor-cell-intrinsic factors that are important for successful metastatic colonization in various target organs [1]. Pioneering work by Hunter and others using mouse genetic crossing has also revealed the critical role of the host genetic background in determining metastatic efficiency [2]. However, genetic screening of host tissue regulators of metastasis has been difficult and rarely attempted previously. In a recent issue of *Nature*, van der Weyden and colleagues used 810 mutant mouse strains to conduct a tour de force in vivo screen of host genes involved in regulating metastatic colonization [3]. The study revealed that deficiency of *Spns2*, which encodes a transporter of sphingosine-1-phoshate (S1P) that regulates lymphocyte trafficking, strongly suppressed lung metastasis colonization by increasing the effector-T-cell- and natural killer (NK)-cell-mediated immune defense present at distant organs.

A major hurdle for successful metastatic colonization of DTCs is the host tissue immune system [1]. It has been shown that cytotoxic T cells are critical for preventing metastatic colonization of melanoma in the lungs [4], and that NK cell depletion increases metastasis [5]. Metastatic cancer cells have been reported to develop mechanisms to suppress the host immune system [1] or evade immune cell killing [6]. Interestingly, previous studies also indicate that host tissue polymorphisms can alter the risk of cancer metastasis by affecting immune surveillance of cancer. For example, *Cadm1* is a metastasis susceptibility locus gene that suppresses metastasis by sensitizing tumor cells to T-cell-mediated killing [7].

In the *Nature* study, the authors tested lung metastasis efficiency of the B16-F10 metastatic mouse melanoma cell line in 810 randomly selected mutant mouse strains that are defective in genes involved in a wide range of biological functions. They identified 23 host mutations that significantly decreased or increased the number of lung metastatic lesions. Interestingly, 19 out of these 23 mutant mouse strains displayed immune-related phenotypes, which implied a prominent involvement of the host immune system in regulating metastatic colonization. Not surprisingly, mutations that caused deficiency in the interferon response, such as loss of the interferon regulatory factor genes *Irf1* and *Irf7*, resulted in an increased incidence of metastasis. On the other hand, mutations in 15 genes, including many that have not previously been implicated in metastasis, reduced the rate of metastasis. The strongest metastatic suppression was observed in the *Spns2* ^*tm1a/tm1a*^ mutant mouse strain. While primary tumor growth was not affected in *Spns2* mutant mice*,* spontaneous and experimental metastasis to lung and liver was reduced when they were injected with metastatic melanoma, colorectal, or breast cancer cell lines. Importantly, although *Spns2* mutation did not affect the initial dissemination and extravasation of cancer cells, an increased number of apoptotic cancer cells were observed in the lung. These findings indicate that host SPNS2 fosters a more favorable environment for the survival of DTCs in the lung.

SPNS2 is a cell surface protein that transports intracellular S1P to blood and lymph, where S1P acts as a bioactive lipid mediator that binds to its G-protein-coupled receptor to regulate cell survival, proliferation, migration, angiogenesis, lymphangiogenesis, lymphocyte trafficking, and immune response [8]. Consistent with the critical role of SPNS2 in S1P transport, *Spns2* ^*tm1a/tm1a*^ mice have lower levels of S1P in serum and increased levels in the lung, which resulted in a profound alteration of leukocyte trafficking in the animals. In addition to a significant reduction of T and B cells in circulation, a dramatic increase in the NK-cell population and a reduced T-cell percentage were observed in the lungs of *Spns2* ^*tm1a/tm1a*^ mice.

The authors used bone marrow transplantation experiments to conclude that a non-hematopoietic stromal component controls the *Spns2-*mediated phenotype in leukocyte trafficking and metastasis*.* As an S1P gradient in lymph has been reported to be crucial for regulating lymphocyte circulation, the researchers focused their investigation on the lymphatic endothelium. Indeed, mice with lymphatic-endothelial-cell-specific deletion of *Spns2* (*Spns2* ^*tm1a/tm1a*^; *Lyve*1^cre/+^) showed decreased lymphocyte counts in the blood, lungs, and other tissues, and decreased metastatic colonization by B16-F10 melanoma cells (Fig. 1). These findings indicate that *Spns2* deficiency in lymphatic endothelium alters the immune microenvironment of the lungs and possibly other organs to reduce metastatic colonization.

**Fig. 1:**
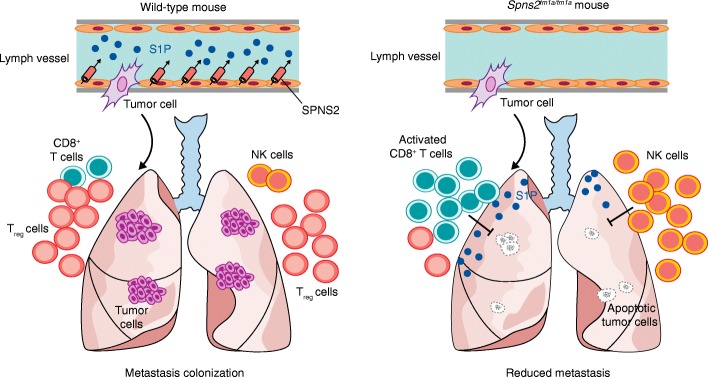
Endothelial SPNS2 regulates lymphocyte trafficking to influence metastatic colonization. After extravasation into the lung parenchyma following dissemination through vascular or lymphatic systems, cancer cells normally encounter a hostile environment dominated by immune defenses. In wild-type mice (*left panel*), the S1P transporter SPNS2 regulates the circulation levels of S1P and maintains lymphocyte trafficking homeostasis as well as organ regulatory T (*T*_*reg*_) cells. In this scenario, the T_reg_ cells are abundant in the lung tissue, which facilitates the colonization of highly metastatic tumor cells. However, in the *Spns2*-deficient mice (*Spns2* ^*tm1a/tm1a*^; *right panel*), S1P levels are decreased in circulation but are higher in the lungs, disrupting lymphocyte trafficking. This results in an increased ratio of cytotoxic CD8^+^ T cells and natural killer (*NK*) cells in the lungs, which prevents metastatic colonization of arriving cancer cells

At first glance, it seems counterintuitive that a decreased lymphocyte count weakens metastatic colonization. However, the authors went on to demonstrate that despite a general decrease in T cells, the ratio of effector T cells and immunosuppressive regulatory T (T_reg_) cells was increased in the lungs of *Spns2-*deficient mice, as were NK cell numbers. In addition, CD4^+^ and CD8^+^ cells from *Spns2* ^*tm1a/tm1a*^ animals showed a stronger degranulation response, increased interferon-γ production, and more effective B16-F10 tumor cell killing in vitro, indicating higher T-cell activity. In vivo T-cell and NK-cell depletion experiments showed that combined depletion of CD8^+^ T cells and NK cells, but not either population alone, restored the metastatic efficiency of cancer cells in *Spns2* ^*tm1a/tm1a*^ mice to the levels observed in wild-type mice. Similar findings were observed in the liver, demonstrating that both T cells and NK cells are responsible for and work cooperatively to provide defense against metastasis in different organs. Finally, treatment of wild-type mice with 4′-deoxypyridoxine (DOP), which inhibits S1P degradation and thus increases S1P levels, led to a similar increase in immune-mediated killing and suppression of lung metastasis as observed in the *Spns2* ^*tm1a/tm1a*^ mice. This result indicates that S1P levels regulate lymphocyte circulation and modulate the percentage of effector T cells and NK cells in the lung, thereby offering an attractive potential therapeutic target for metastatic colonization (Fig. 1).

While previous research in the field had revealed intricate networks of tumor–stromal interactions during metastasis, the current study represents the first extensive in vivo screen of host factors that influence metastatic colonization. Several other genes identified in the screen still remained uncharacterized for their functional mechanism in metastasis, representing many additional avenues for future explorations. Further efforts on similar in vivo functional screens should focus on “druggable” classes of genes with no life-threatening phenotypes in mutant mice, as these genes are likely ideal targets for therapeutic intervention.

Consistent with the findings observed in mouse models, *SPNS2* has been reported to be upregulated in the stromal gene expression signature associated with a poor clinical outcome of human breast cancer [9], which underscores the clinical relevance of the present study. The identification of SPNS2 as a novel regulator of the host immune response to metastasis further highlights the essential role of resident immune cells in guarding and protecting against metastasis. The relevance of this mechanism is particularly salient, as immunotherapy has been proven to have striking effects in metastatic cancer patients, especially in those with metastatic melanoma [10]. It is unclear why SPNS2 deficiency specifically affects the development of metastases but has no impact on primary tumor growth. It is possible that initial metastatic seeding is particularly sensitive to immune clearance, while established tumors are more refractory to the attack of effector T cells and NK cells. Alternatively, the immune microenvironment in the lung and other organs may be more responsive to S1P levels. Future studies need to investigate these questions, and explore the therapeutic effect of S1P and SPNS2 inhibition in established metastasis, which more closely mimics the clinical situation of late-stage cancer patients.

There are several potential translational applications of these new insights into SPNS2 and S1P as functional regulators of metastasis. As circulating S1P levels are tightly controlled by SPNS2, and increasing S1P levels with DOP treatment can suppress metastasis, S1P could be used as a biomarker of metastasis susceptibility in cancer patients. Such analysis should be performed after stratifying patients based on their cancer subtypes, as varying degrees of immune infiltration have been reported in different subtypes of breast cancer and other cancers. Targeting S1P using blocking antibodies such as sphingomab could be complicated by potential side effects in the immune and vascular systems. Alternatively, because it is a cell surface transporter, neutralizing antibodies or small inhibitors against SPNS2 could be developed as agents to prevent or reduce metastasis. In addition, genetic polymorphism, somatic mutations, or other means of increased expression or activity of SPNS2 and other components of the S1P pathway may play a causal role in promoting cancer metastasis, which should be an important topic for future investigations. Overall, this study represents a novel approach to study the complicated role of host tissue in cancer metastasis and opens up a potential new avenue to increase the efficacy of immunotherapy for metastatic cancer.

## Abbreviations

DOP: 4′-Deoxypyridoxine
DTC: Disseminated tumor cells
NK: Natural killer
T_reg_: Regulatory T cell
